# Diversity of Phototrophic Genes Suggests Multiple Bacteria May Be Able to Exploit Sunlight in Exposed Soils from the Sør Rondane Mountains, East Antarctica

**DOI:** 10.3389/fmicb.2016.02026

**Published:** 2016-12-19

**Authors:** Guillaume Tahon, Bjorn Tytgat, Anne Willems

**Affiliations:** Laboratory of Microbiology, Department of Biochemistry and Microbiology, Ghent UniversityGhent, Belgium

**Keywords:** Princess Elisabeth Station, Sør Rondane Mountains, anoxygenic phototrophic bacteria, actinorhodopsin, light-harvesting, AAP

## Abstract

Microbial life in exposed terrestrial surface layers in continental Antarctica is faced with extreme environmental conditions, including scarcity of organic matter. Bacteria in these exposed settings can therefore be expected to use alternative energy sources such as solar energy, abundant during the austral summer. Using Illumina MiSeq sequencing, we assessed the diversity and abundance of four conserved protein encoding genes involved in different key steps of light-harvesting pathways dependent on (bacterio)chlorophyll (*pufM, bchL*/*chlL*, and *bchX* genes) and rhodopsins (actinorhodopsin genes), in exposed soils from the Sør Rondane Mountains, East Antarctica. Analysis of *pufM* genes, encoding a subunit of the type 2 photochemical reaction center found in anoxygenic phototrophic bacteria, revealed a broad diversity, dominated by *Roseobacter*- and *Loktanella*-like sequences. The *bchL* and *chlL*, involved in (bacterio)chlorophyll synthesis, on the other hand, showed a high relative abundance of either cyanobacterial or green algal trebouxiophyceael *chlL* reads, depending on the sample, while most *bchX* sequences belonged mostly to previously unidentified phylotypes. Rhodopsin-containing phototrophic bacteria could not be detected in the samples. Our results, while suggesting that Cyanobacteria and green algae are the main phototrophic groups, show that light-harvesting bacteria are nevertheless very diverse in microbial communities in Antarctic soils.

## Introduction

Antarctica is nearly completely covered by ice, with only ~0.32% of its surface ice-free. Although most of the ice-free regions are found in the Antarctic Peninsula and the Transantarctic Mountains, inland mountain ranges, such as the Sør Rondane Mountains (Dronning Maud Land), also represent an important fraction of the exposed surface area (Convey et al., 2008; Cary et al., 2010). The absence of vascular plants in continental Antarctica combined with the extreme environmental conditions have led to depleted soils with low availability of nutrients, especially of organic carbon, and nitrogen, and water (Kennedy, 1993; Cary et al., 2010; Tytgat et al., 2016). As a result, the mainly microscopic life in these areas (Cowan et al., 2014; Tytgat et al., 2014; Obbels et al., 2016) may thus be expected to use alternative energy sources to overcome these limitations. Sunlight, abundantly available during the austral summer, may be an important resource for certain members of the bacterial communities inhabiting exposed continental environments, and this should be reflected in the diversity of key genes for light-harvesting functions.

As life on Earth evolved, microorganisms developed different ways to harvest solar energy. Two main mechanisms have been described, either using rhodopsins or complex photochemical reaction centers that contain (bacterio)chlorophyll (Bryant and Frigaard, 2006). Early phototrophic prokaryotes (~3.5 Giga annum ago) used reductants such as H_2_, Fe^2+^, or H_2_S for bacteriochlorophyll-dependent anaerobic anoxygenic phototrophy and did not involve oxygen (Hohmann-Marriott and Blankenship, 2011; Butterfield, 2015; Cardona, 2016). Later on (at least ~2.4 Giga annum ago), oxygenic chlorophyll-dependent phototrophy, using H_2_O, arose in Cyanobacteria and played a key role in oxygenating the Earth's atmosphere (Butterfield, 2015; Cardona, 2016). Under these new atmospheric conditions, many of the anaerobic anoxygenic phototrophic bacteria may have disappeared from the now oxygenated habitats, although some groups adapted and embarked on an aerobic lifestyle (Koblížek, 2015). These aerobic anoxygenic phototrophic bacteria (AAP) were first reported in 1978 (Harashima et al., 1978) and are defined as aerobic species that synthesize bacteriochlorophyll and use light energy as an auxiliary energy source for their mostly heterotrophic metabolism (Feng et al., 2011a; Koblížek, 2015). Moreover, they do not contain carbon fixation enzymes (Yurkov and Csotonyi, 2009). Since their discovery nearly four decades ago, numerous AAP, predominantly belonging to the Proteobacteria, have been described from various habitats (Koblížek, 2015). Some species capable of aerobic anoxygenic phototrophy have also been found in the Gemmatimonadetes, Acidobacteria, and Chloroflexi. Phototrophic species belonging to the latter phylum are, however, not included in the AAP, as are many other aerobic bacteria that synthesize Bchl and perform anoxygenic phototrophy under aerobic conditions (e.g., phototrophic methylotrophs, Yurkov and Csotonyi, 2009; Zeng et al., 2014; Koblížek, 2015). The majority of all aforementioned anoxygenic phototrophs rely on a heterodimeric type 2 reaction center with *pufL* and *pufM* encoding the conserved proteins. Hence, these two *puf* genes are frequently used and convenient markers to study the diversity of anoxygenic phototrophic bacteria (Koh et al., 2011; Ritchie and Johnson, 2012; Koblížek, 2015).

Additionally, several other genes encoding subunits of key enzymes in the (bacterio)chlorophyll synthesis pathway are also well conserved among phototrophic microorganisms. All oxygenic and anoxygenic phototrophic bacteria use the dark-operative protochlorophyllide oxidoreductase (DPOR) enzyme complex, encoded by the *chlLNB* and *bchLNB* genes, respectively. Apart from in bacteria, DPOR is also found in green algae and lower land plants (Nomata et al., 2014). The complex plays a key role in the biosynthesis of (bacterio)chlorophyll, converting protochlorophyllide to chlorin (Fujita and Bauer, 2003; Gupta, 2012). Whereas, in Cyanobacteria, green algae and lower land plants, chlorin is converted immediately to chlorophyll (Chl), in anoxygenic phototrophic bacteria (APB) a second enzyme complex, chlorin oxidoreductase (COR), encoded by *bchXYZ* genes, reduces chlorin to bacteriochlorophyllide, the direct precursor for bacteriochlorophyll (Bchl) (Beale, 1999; Chew and Bryant, 2007). DPOR and COR exhibit a high degree of structural similarity. Interestingly, the amino acid sequences of the different DPOR and COR subunits (BchLNB/ChlLNB and BchXYZ, respectively), exhibit significant similarity (~15–30%) to those of the nitrogenase enzyme complex (NifHDK), leading to the hypothesis that these three enzyme complexes all evolved from the same common ancestor (Fujita and Bauer, 2003; Reinbothe et al., 2010; Gupta, 2012).

Besides phototrophy using photochemical reaction centers, a second type of phototrophy, employing rhodopsins also evolved (Bryant and Frigaard, 2006), although little is known about its origin in time. Microbial rhodopsins have been described in various groups, mostly in aquatic habitats, performing a range of functions, including light-driven ion pumping (Béjà and Lanyi, 2014; Boeuf et al., 2015). Although previously detected in Siberian permafrost (Petrovskaya et al., 2010), Antarctic sea ice, sea water, and continental lakes (Béjà et al., 2001; Koh et al., 2010; Qin et al., 2012; Williams et al., 2012; Yau et al., 2013; Do et al., 2014; Markowitz et al., 2014), little rhodopsin data are available for terrestrial Antarctica (based on metagenome data available on MG-RAST Wilke et al., 2016 and IMG Markowitz et al., 2014). Recently, a new family of proton pumping microbial rhodopsins, actinorhodopsins, has been discovered in freshwater Actinobacteria, (Sharma et al., 2008). To our knowledge, their occurrence in Antarctica has not been reported yet.

A first cloning survey of genes for phototrophic mechanisms in samples from the oligotrophic high-altitude soils near the Belgian Princess Elisabeth Station in the Sør Rondane Mountains revealed a high diversity of *pufLM* genes, whereas proteorhodopsin genes could not be amplified from any of the samples (Tahon et al., 2016). In this study, we aimed to more comprehensively assess the diversity of bacteria capable of exploiting sunlight as an alternative energy source. To further test the hypothesis that sunlight may be a very important resource for certain members of the bacterial communities inhabiting these exposed oligotrophic soils, an Illumina MiSeq paired-end 300 bp sequencing approach was used with primers targeting *pufM*, actinorhodopsin and *bchL*/*chlL*/*bchX* genes. For the latter, sequence data obtained in a previous study into the diversity of *nifH* genes (Tahon et al., under review), that were originally discarded because they lacked multiple of the conserved NifH amino acid residues, were revisited. Further analyses reported here identified these sequences as the NifH homologs BchL/ChlL and BchX, involved in the (bacterio)chlorophyll synthesis pathways (Fujita and Bauer, 2003; Raymond et al., 2004).

## Materials and methods

### Study site and sample collection

Four samples, previously used in the pilot survey (Tahon et al., 2016), were studied (Table 1), to allow comparison. During the Antarctic summer of 2009, top surface samples—mostly consisting of weathered granite parent material—were collected aseptically in the vicinity of the Belgian Princess Elisabeth Station (71° 57′ S, 23° 20′ E) at Utsteinen, Dronning Maud Land, East Antarctica. All samples were frozen at −20°C upon collection. Sample KP2 was collected ~1.3 km south of the research station. The three other samples were collected on the Utsteinen ridge, ~500 m north of the Belgian base.

**Table 1:** **Parameters associated with analyzed samples**.

**Sample**	**Sample coordinates**	**Altitude (m)**	**Description of sample area**	**Conductivity (μS/cm)**	**pH**	**Water content**	**TOC**
KP2	71° 57' 28.6″ S, 23° 19' 45.8″ E	1320	Small gravel particles in between rocks, Utsteinen nunatak	19	6.54	6.28%	0.08%
KP15	71° 56' 45.8″ S, 23° 20' 43.6″ E	1366	Brown soil under lichen, East part of Utsteinen ridge	33	5.57	3.38%	0.33%
KP43	71° 56′ 47.3″ S, 23° 20′ 44.6″ E	1362	Brown soil with dark green fragments, East part of Utsteinen ridge	520	6.22	0.91%	2.57%
KP53	71° 56′ 45.3″ S, 23° 20′ 42.4″ E	1362	Grey soil on East part of Utsteinen ridge	312	6.34	0.23%	0.21%

### DNA extraction

From each homogenized sample, 400 mg subsamples were taken in triplicate. Total genomic DNA was extracted and purified using the PowerLyzer® PowerSoil® DNA isolation kit (MoBio Laboratories) and a modified lysis protocol as instructed by the manufacturer. This extraction protocol was previously identified as the one yielding most bacterial diversity (Tahon et al., 2016). Following extraction, DNA was quantified using the Qubit® 2.0 fluorometer (Life Technologies) and stored at −20°C until processing.

### PCR and preparation for illumina sequencing

A Veriti thermal cycler (Life Technologies) was used to amplify partial actinorhodopsin, *pufM, bchL*/*chlL*, and *bchX* genes. Primer selection was based on two criteria: (1) to amplify a broad diversity of the gene and (2) to produce an amplicon size suitable for Illumina MiSeq 300 bp paired-end sequencing (Table 2). To complement the Nextera XT index kit (Illumina), primers were extended with an adapter.

**Table 2:** **PCR primers (without adapters) and conditions used for screening different genes**.

**Gene**	**Target**	**Primer**	**Sequence 5′-3′**	**Final concentration**	**Region**	**Amplicon size**	**Program^g^**
*pufM*	Universal	pufM_uniF^a^	GGN AAY YTN TWY TAY AAY CCN TTY CA	1.0 μM	584–825^d^	± 240 bp	94°C (4 min); 35x 94°C (40 s), 49°C (30 s), 72°C (30 s); 72°C (7 min)
		pufM_WAW^a^	AYN GCR AAC CAC CAN GCC CA	0.5 μM			
actinorhodopsin	Clade LG1 & LG2	LG-for^b^	TAY MGN TAY GTN GAY TGG	0.4 μM	283–614^e^	± 330 bp	95°C (7 min), 45x 94°C (30 s), 51.5°C (1 min 30 s), 72°C (30 s); 72°C (10 min)
		LG1A-for^b^	MGN TAY ATH GAY TGG YT	0.4 μM			
		LG2-for^b^	TAY MGN TAY GCN GAY TGG	0.4 μM			
		LG-rev^b^	ATN GGR TAN CAN CCC CA	0.8 μM			
*nifH, bchL, chlL, bchX*	Universal	IGK3^c^	GCI WTH TAY GGI AAR GGI GGI ATH GGI AA	1.0 μM	19–413^f^	395 bp	95°C (10 min); 40x 95°C (45 s), 52°C (30 s), 72°C (40 s); 72°C (10 min)
		DVV^c^	ATI GCR AAI CCI CCR CAI ACI ACR TC	1.0 μM			

a From (Yutin et al., 2005)

b From (Sharma et al., 2009)

c From (Ando et al., 2005)

d Based on the pufM sequence of Sphingomonas sanxanigenens DSM 19645 (CP006644)

e Based on the actinorhodopsin sequence of Leifsonia rubra CMS 76R (ATIA01000023)

f Based on the nifH sequence of Azotobacter vinelandii (M20568)

g *All programs were optimized in this study*.

For each of the soil samples, PCR was performed in triplicate on all three DNA extracts, for each primer set, resulting in a total of nine PCR products per sample per gene. PCRs were performed in 25 μl reaction mixtures containing 3 μl of genomic DNA (>6 ng μl^−1^), 1x Qiagen PCR buffer (Qiagen), 0.2 mM of each deoxynucleotide triphosphate, 0.625 U of Qiagen *Taq* polymerase (Qiagen), 100 mM bovine serum albumin and forward and reverse primer with final concentrations as shown in Table 2. All nine PCR products (three DNA extracts x three PCRs) were pooled and purified using the Ampure beads XT (Agencourt) protocol with slight modifications. Briefly, only 0.8 reaction volume of beads was used and DNA was resuspended in MilliQ water. Tagging of pooled PCR products was performed using the Nextera XT indices (Illumina) during an eight cycle version of the amplicon PCR with the indices replacing the primers. Afterwards, PCR products were purified as described above, with resuspension in Tris buffer (0.1 M, pH 8.5). Integrity and amplicon sizes of the PCR products were checked using a BioAnalyzer (Agilent), following quantification using a Qubit, as described above. Afterwards, samples were pooled equimolarly and sequenced on an Illumina MiSeq 300 bp paired-end platform (GATC). PhiX was spiked at 20% per lane.

### Sequence data processing

For all genes, the forward and reverse sequencing reads were merged using the *fastq_mergepairs* command in USEARCH (Edgar, 2010) allowing a minimum overlap length of 8 nucleotides and a maximum of six mismatches in the overlapping region. For *bchL*/*chlL*/*bchX*, and *pufM*, merged sequences shorter than 370 and 200, and longer than 470 and 350, were removed using the *fastq_minmergelen* and *fastq_maxmergelen* commands, respectively. Primer sequences were removed from the merged sequences using cutadapt v1.8 (Martin, 2011), resulting in sequences with a minimum length of 193 and 321 bp, and a maximum length of 225 and 369 bp for *pufM* and *bchL*/*chlL*/*bchX*, respectively. Subsequently, during quality filtering using USEARCH, sequences with one or more nucleotides beneath the Phred Q20 threshold score and a maximum error >0.5 were removed from further analyses. Afterwards, all sequences were placed in reading frame +1, followed by removal of sequences showing no similarity to our genes of interest or containing stop codons and/or indels resulting in a frameshift. Detection of putative chimeric sequences was done using the Uchime model (default parameters, Edgar et al., 2011) in Mothur (Schloss et al., 2009). Finally, all remaining sequences were translated to proteins using MEGA 6 using the bacterial genetic code (Tamura et al., 2013).

### Sequence analyses

For *pufM*, an updated version of our previously described database containing publicly available sequences (Tahon et al., 2016) was used. For *bchL*/*chlL* and *bchX* a new database was assembled to contain all related sequence records from NCBI and IMG (https://img.jgi.doe.gov/) (Markowitz et al., 2012) available per November 15th 2015. Newly obtained nucleotide sequences and their derived protein sequences were added to the databases using the import module of BioNumercs 7.5 (Applied Maths). For the sequences obtained with the primer set IGK3/DVV, to retain only BchL/ChlL (L subunit of DPOR) and BchX (X subunit of COR) sequences, NifH sequences were separated based on the presence of the conserved amino acid residues Ala43, the dipeptide Glu93-Pro94, Arg101, the dipeptide Ile104-Thr105 and Glu111 (positions based on the NifH protein sequence of *Azotobacter vinelandii*, accession number M20568, Fujita and Bauer, 2003). For phylogenetic analyses, all Illumina PufM sequences were clustered at a 95% cut-off using CD-HIT (Li and Godzik, 2006; Fu et al., 2012), grouping them into operational puf units (OPUs) of which one representative was used to construct the phylogenetic tree. BchL/ChlL and BchX sequences were grouped in operational BchL/ChlL units (OLUs) and operational BchX units (OXUs), respectively, at 95% cut-off. BchL/ChlL and BchX sequences were processed together for phylogenetic analyses. A first alignment was made with all sequences present in our databases, using Clustal Omega (Goujon et al., 2010; Sievers et al., 2011). Afterwards, alignments were trimmed to the size of our sequenced amplicons and visually inspected, excluding from further analysis all non-overlapping reference sequences. Remaining sequences were realigned, after which the alignment was used to construct a maximum likelihood (ML) phylogenetic tree (1000 bootstrap replicates), by using the FastTree tree building software (Price et al., 2010) with the Whelan and Goldman evolutionary model and the discrete gamma model with 20 rate categories. From the resulting phylogenetic trees, closest relatives of our newly obtained sequences as well as representative sequences from the entire tree were selected to prepare a smaller tree representing the initial complete tree, following the same protocol. Sequences from uncultured bacteria were not included in the final tree. Trees were visualized using the iTOL software (Letunic and Bork, 2007, 2011) and related OPUs, OLUs, or OXUs were grouped into visual clusters that were named after cultivated bacteria that grouped in or close to the cluster. In the absence of cultivated members, the clusters were given an Utsteinen (UT) cluster number designation.

### Statistical analyses

For statistical analyses of PufM, BchL/ChlL, and BchX sequences, the Vegan package (Dixon, 2003) in R (https://cran.r-project.org/) was used. A non-normalized table of the total number of protein sequences per OPU, OLU, or OXU was used to perform rarefaction analyses and determine the total number of expected OPUs/OLUs/OXUs per sample and for the four samples combined. Parameters calculated for each dataset include species richness (Chao1) and evenness (Pielou). A normalized table (consensus of 10,000 iterations) of the total number of protein sequences per OPU, OLU, or OXU was also generated, to assess the impact on relative abundances.

### Accession numbers

Raw sequences were submitted to the NCBI sequence read archive under accession number SRP067116.

## Results

Actinorhodopsin genes could not be amplified from the samples and were therefore not included in the Illumina run. Sequencing and thorough quality control of sequence data resulted in 678940 high-quality *pufM* sequences (length 193–198 bp) and 119822 and 4950 high-quality *bchL*/*chlL* and *bchX* sequences (length 321–348 bp). At a 95% cut-off, the PufM protein sequences constituted 925 OPUs of which 248 were singletons. For BchL/ChlL and BchX, the sequences grouped in 207 OLUs and 48 OXUs, respectively. A total of 48 OLUs and 18 OXUs were singletons (Table 3).

**Table 3:** **Overview of sequence data characteristics**.

		**Illumina data**					**Normalized data**				
		**KP2**	**KP15**	**KP43**	**KP53**	**All**	**KP2**	**KP15**	**KP43**	**KP53**	**All**
PufM	No. of sequences	192517	246511	95721	144191	678940	11715	11715	11715	11715	46860
	No. of OPUs	509	644	502	463	925	72	85	110	71	171
	No. of singleton OPUs	44	97	76	31	248	6	18	35	15	74
	No. of identified singleton OPUs^a^	43	92	76	31	242	6	16	35	15	72
	evenness (*H*/*H*_max_)	0.106	0.128	0.261	0.100	0.136	0.141	0.171	0.331	0.130	0.188
	Chao1	666.51	884.32	781.40	634.92	1227.39	110.75	214.00	157.83	166.14	329.88
BchL/ChlL	No. of sequences	72910	18836	11715	16361	119822	11715	11715	11715	11715	46860
	No. of OLUs	115	113	75	66	207	82	113	75	66	192
	No. of singleton OLUs	11	13	17	7	48	7	22	17	9	55
	No. of identified singleton OLUs^a^	11	13	16	7	47	7	22	16	9	54
	evenness (*H*/*H*_max_)	0.267	0.243	0.252	0.214	0.380	0.286	0.244	0.252	0.214	0.395
	Chao1	136.11	130.65	120.11	85.25	277.50	124.86	158.56	120.11	154.00	279.35
BchX	No. of sequences	53	345	4241	311	4950					
	No. of OXUs	7	19	32	8	46					
	No. of singleton OXUs	3	5	10	0	18					
	No. of identified singleton OXUs^a^	3	5	10	0	18					
	evenness (*H*/*H*_max_)	0.623	0.315	0.168	0.216	0.231					
	Chao1	13.00	30.25	45.75	8.33	67.86					

*Diversity indices were calculated on the basis of derived protein sequences, binned at 95% similarity*.

a *The number of identified singleton OPUs, OLUs or OXUs corresponds to singletons that show a high similarity to, respectively, PufM, BchL/ChlL or BchX sequences of cultured organisms or that belong to the Utsteinen (UT) clusters (details provided in Table S1)*.

When analyzing sequencing data, there is no consensus on inclusion or removal of singletons. Although many authors choose to remove them, particularly when the focus is on dominant community members (Pedrós-Alió, 2012), we have opted not to do so, for several reasons. Previous research has shown that singletons may be informative and valuable in reflecting rare and/or unique lineages of dormant or inactive bacteria that may grow when the right conditions are met (Logares et al., 2014). Removal of singletons would thus lead to a loss of power to detect these rare lineages in communities and lead to an underestimation of biodiversity levels (Kauserud et al., 2012; Zhan et al., 2013, 2014; Pascual et al., 2016). On the other hand, singletons may represent erroneous sequences and therefore the use of a very stringent quality control is required to accurately sort informative low-abundance sequence reads from errors and artifacts. We therefore implemented a very stringent quality control. The length of the *pufM, bchX*, and *bchL*/*chlL* amplicons (Table 2) allowed a large or even complete overlap between the forward and reverse sequencing reads. In the overlapping region, only six mismatches were allowed and every sequence with one or more nucleotides with a base call accuracy lower than 99% was discarded. Furthermore, since we studied protein-encoding genes rather than 16S rRNA genes, a number of additional quality control steps could be performed. The gene sequences were placed in frame +1, translated into amino acids and these were analyzed for the presence of stop codons, indels that result in a frameshift and presence of conserved sites. These steps allowed additional removal of erroneous sequences so that the number of singleton sequences was reduced a thousand fold and the final data are of much higher quality and likely represent real, though perhaps rare, diversity. Indeed, the vast majority of leftover singletons were found to group within the named clusters or close to sequences of named species (**Figures 3**, **4A,B**, and Table 3, Table S1). As the goal of our study was to explore the (nearly) whole diversity of several protein encoding genes, including rare types, the number of singletons retrieved also aids to get more insight in the diversity coverage of the approach used. To estimate total diversity in a sample different parameters (e.g., Chao1, ACE) can be used (Gotelli and Colwell, 2010, Table 3). Calculation of these parameters takes the number of singletons into account and removal of singletons would confound the ability to estimate alpha diversity.

**Figure 1:**
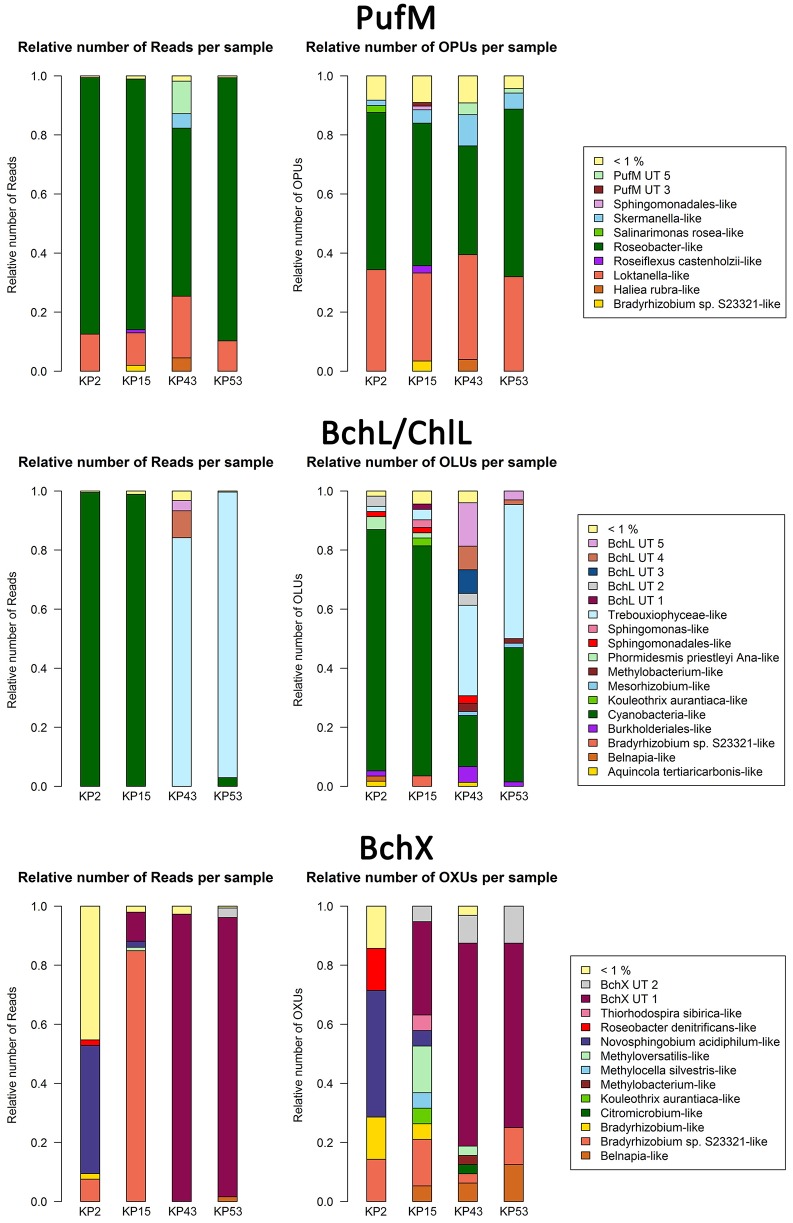
**Bar plots showing relative numbers of reads and OPUs (PufM), OLUs (BchL/ChlL) or OXUs (BchX) per cluster**. Data were not normalized (normalized bar plots are shown in Figure S2). Clusters or separate OPUs/OLUs/OXUs containing less than 1% of the data were grouped together in the <1% group.

As there is no consensus on the use of normalization which may lead to the omission of valuable diversity data (McMurdie and Holmes, 2014), we assessed how much diversity would be lost by normalizing. For each gene, the original OTU table was normalized 10,000 times. Based on these results, box plots were generated showing the large variation in the OTU diversity recovered after standardization (Figure S1). Therefore, we created a consensus OTU table out of 10,000 standardizations and used this for calculating the relative abundances of reads and OTUs (Figure S2). Surprisingly, differences with relative abundances calculated using non-normalized data (Figure 1) are limited (<0.05% for most of the OTUs and clusters). Normalization does, however, involve removal of sequence data and as a result leads to the discarding of (rare) diversity. This is most clear in the BchX dataset where the effect of normalization on sample KP43 is more pronounced as this sample had a very large number of reads and rare OTUs, many of which were removed. To avoid potential loss of rare sequence types, non-normalized data was used in the diversity assessments.

**Figure 2:**
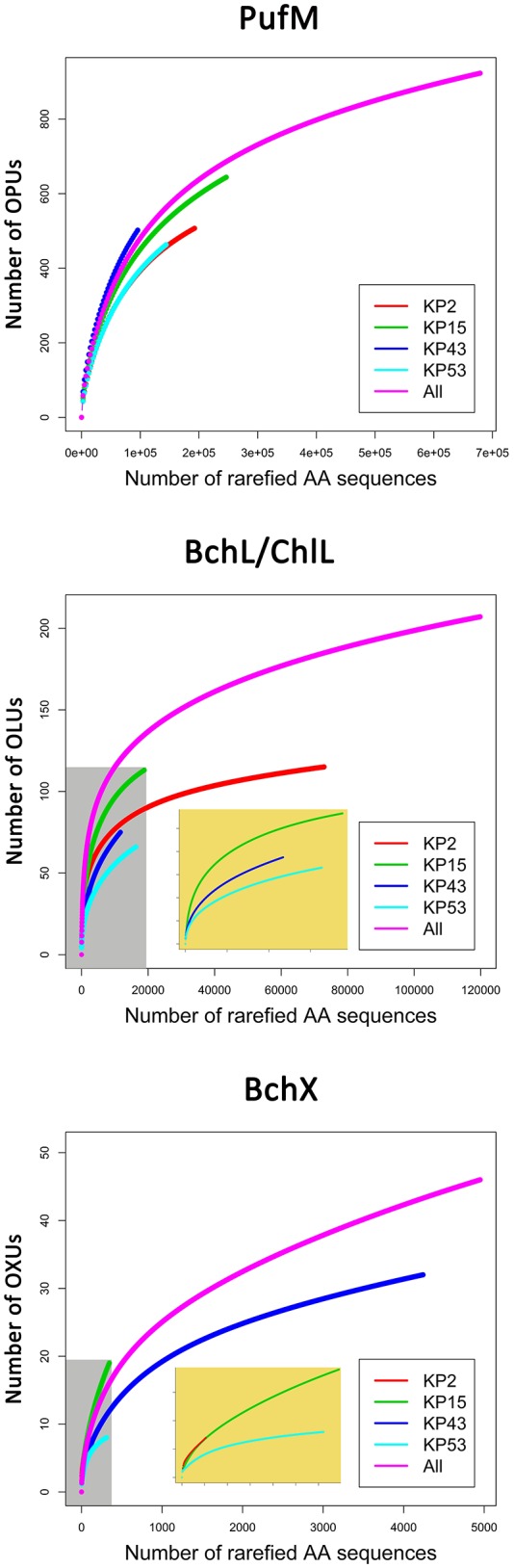
**Rarefaction curves based on grouping protein sequences that have 95% similarity**. Analysis was performed using the Vegan package in R. Embedded figures (beige background in BchL/ChlL and BchX) show a more detailed view of rarefaction curves completely enclosed in the gray area.

For PufM, of the 925 OPUs, 358 were detected in one sample only (Figure S3, Table S1). The number of OPUs in each sample varied from 463 to 644 (Table 3). Of 228 shared OPUs, two contained the majority of PufM sequences and both grouped with PufM of heterotrophic alphaproteobacterial AAPs. OPU C1 contained 80.11% of all sequences and was 98.48% similar to the PufM sequence of *Roseobacter denitrificans* OCh 114 (Figure 1, Table S1). OPU C2 comprised 12.03% of all reads and had the PufM sequence of *Loktanella* sp. RCC2403 as closest match (96.97% amino acid similarity) (Figure 1, Table S1). Rarefaction analysis showed that, although the graphs started to flatten, saturation was not yet reached (Figure 2). This was corroborated by the number of estimated OPUs (Chao1) that was much higher than the number of observed OPUs (Table 3).

**Figure 3:**
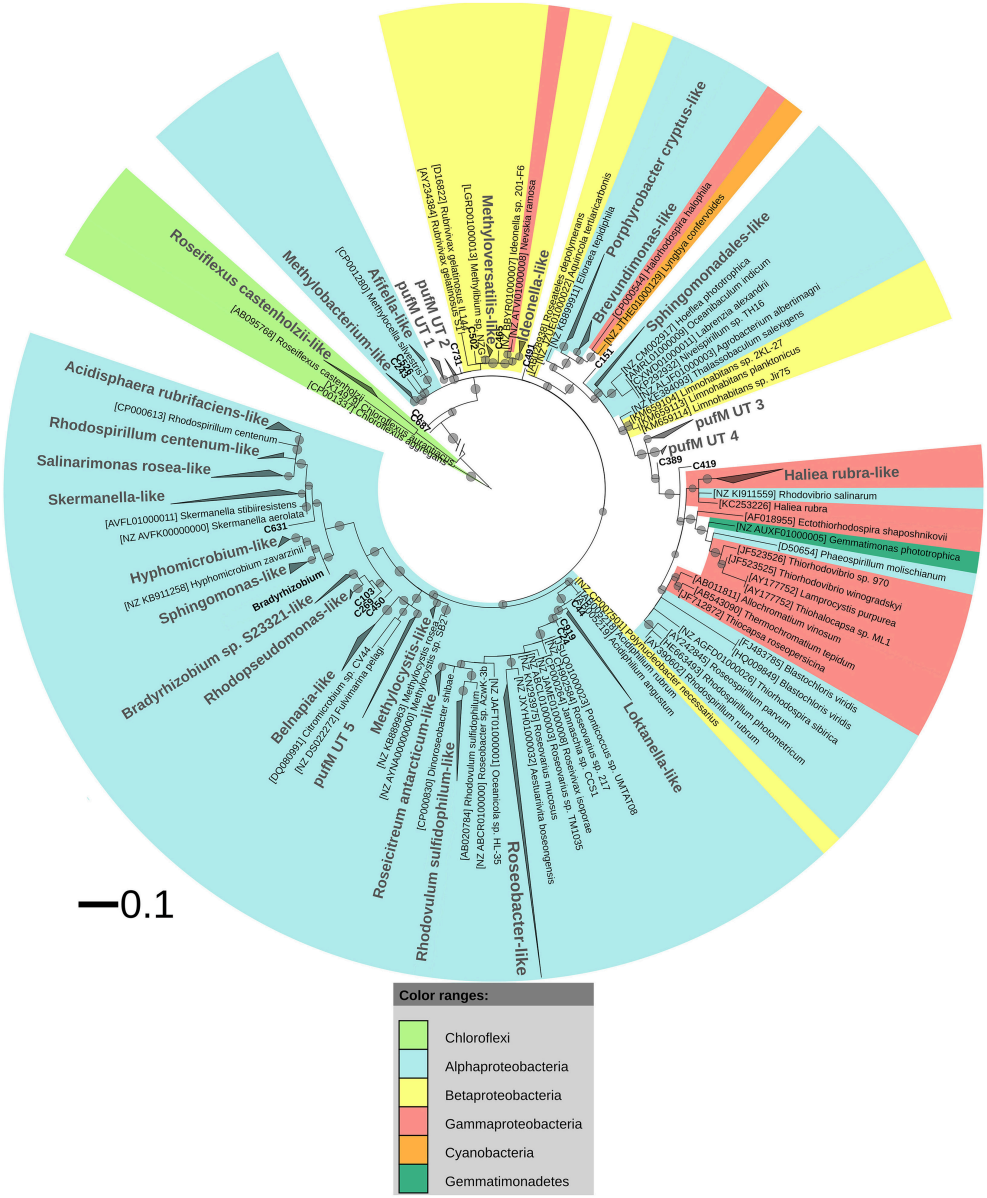
**ML phylogenetic tree (1000 bootstrap replicates) of PufM sequences**. Scale bar indicates 0.1 substitutions per amino acid position. OPU clusters (larger font size) were named after cultivated bacteria that grouped in or close to the cluster. In the absence of cultivated members, clusters were given an Utsteinen (UT) cluster number designation. For clusters, the total branch lengths to the closest and farthest leaf of the cluster were used as sides of the triangle. OPUs not enclosed in clusters are labeled in bold. For reference data, taxon name, and accession number is listed. Bootstrap values are displayed as circles with a diameter reflecting the height of the bootstrap value. Smallest circles represent the lower cut-off of 70%. Chloroflexi PufM sequences were used as an outgroup. PufM sequences originating from cultured *Bradyrhizobium* species were grouped in the cluster defined as “*Bradyrhizobium*” to simplify the topology of the tree.

After ML analysis, nearly all 925 OPUs grouped into 28 clusters (Figure 3), mostly containing reads from all four terrestrial samples as well as PufM sequences originating from cultured bacteria (Table 4). The *Loktanella*-like and *Roseobacter*-like clusters were the largest, containing 273 and 386 of the OPUs and, since they contained OPUs C2 and C1, they also contained most of the reads: 12.70 and 82.39% respectively. It should be noted that the cluster defined as *Roseobacter*-like also contains some PufM sequences of *Tateyamaria, Erythrobacter*, and *Jannaschia* (<0.1% of reads). The third largest cluster (PufM UT 5) grouped among less related alphaproteobacterial AAP PufM sequences and contained only 1.59% of the sequence data, mostly originating from sample KP43 (Figures 1, 3, Table 4). The other clusters and the separate OPUs each contained less than 0.85% of the reads. In the phylogenetic ML tree, our OPUs grouped with a broad variety of known PufM sequences originating from Alpha-, Beta-, and Gammaproteobacteria, and even Chloroflexi (Figure 3). Affiliations with alphaproteobacterial AAP PufM sequences, however, were most frequent. Several clusters (PufM UT 1–PufM UT 5) and separate OPUs did not group closely with known diversity, suggesting that many organisms harboring *pufM* genes still remain unreported.

**Table 4:** **Distribution of OTUs (95% protein similarity) and reads per PufM, BchL/ChlL, or BchX cluster or separate OTU**.

	**Cluster/Separate OTU**	**No. of OTUs**	**OTUs (%)**	**No. of reads**	**Reads (%)**
PufM	*Roseobacter*-like	386 (271, 311, 185, 263)	41.73%	559360 (167181, 209233, 54446, 128500)	82.39%
	*Loktanella*-like	273 (175, 192, 178, 148)	29.51%	86210 (24202, 27177, 20025, 14806)	12.70%
	*Skermanella*-like	74 (9, 29, 53, 25)	8.00%	5796 (153, 600, 4713, 330)	0.85%
	*Haliea rubra*-like	23 (1, 4, 20, 2)	2.49%	4348 (6, 29, 4300, 13)	0.64%
	PufM UT 5	22 (4, 6, 20, 7)	2.38%	10789 (29, 80, 10500, 180)	1.59%
	*Bradyrhizobium* sp. S23321-like	22 (1, 22, 1, 1)	2.38%	4945 (50, 4887, 5, 3)	0.73%
	*Roseiflexus castenholzii*-like	16 (2, 16, 3, 1)	1.73%	2572 (29, 2536, 6, 1)	0.38%
	*Salinarimonas rosea*-like	13 (12, 4, 2, 0)	1.41%	315 (289, 22, 4, 0)	0.046%
	*Sphingomonadales*-like	10 (3, 8, 4, 0)	1.08%	64 (6, 46, 12, 0)	0.0094%
	*Porphyrobacter cryptus*-like	9 (3, 3, 5, 3)	0.97%	671 (82, 17, 453, 119)	0.099%
	PufM UT 3	8 (3, 8, 2, 0)	0.86%	647 (164, 463, 20, 0)	0.095%
	PufM UT 2	6 (1, 5, 5, 1)	0.65%	1209 (8, 775, 419, 7)	0.18%
	*Methylobacterium*-like	6 (1, 2, 5, 2)	0.65%	529 (44, 20, 455, 10)	0.078%
	*Belnapia*-like	6 (1, 0, 4, 4)	0.65%	131 (20, 0, 88, 23)	0.019%
	*Rhodovulum sulfidophilum*-like	6 (1, 5, 1, 1)	0.65%	35 (1, 32, 1, 1)	0.0052%
	*Roseicitreum antarcticum*-like	5 (4, 3, 1, 2)	0.54%	185 (31, 91, 14, 49)	0.027%
	PufM UT 1	5 (0, 1, 4, 0)	0.54%	5 (0, 1, 4, 0)	0.00074%
	PufM UT 4	3 (3, 1, 0, 0)	0.32%	66 (58, 8, 0, 0)	0.0097%
	*Methylocystis*-like	2 (1, 2, 1, 0)	0.22%	276 (2, 177, 97, 0)	0.041%
	*Hyphomicrobium*-like	2 (2, 1, 1, 0)	0.22%	71 (64, 4, 3, 0)	0.010%
	*Ideonella*-like	2 (2, 1, 1, 0)	0.22%	65 (5, 4, 56, 0)	0.0096%
	*Rhodospirillum centenum*-like	2 (1, 2, 0, 0)	0.22%	32 (15, 17, 0, 0)	0.0047%
	*Methylibium*-like	2 (0, 2, 1, 0)	0.22%	10 (0, 8, 2, 0)	0.0015%
	*Acidisphaera rubrifaciens*-like	1 (0, 0, 1, 0)	0.11%	13 (0, 0, 13, 0)	0.0019%
	*Methyloversatilis*-like	1 (0, 1, 0, 0)	0.11%	5 (0, 5, 0, 0)	0.0007%
	*Brevundimonas*-like	1 (1, 0, 0, 0)	0.11%	3 (3, 0, 0, 0)	0.00044%
	*Afifella*-like	1 (0, 1, 0, 0)	0.11%	2 (0, 2, 0, 0)	0.00029%
	*Rhodopseudomonas*-like	1 (1, 0, 0, 0)	0.11%	2 (2, 0, 0, 0)	0.00029%
	*Sphingomonas*-like	1 (0, 1, 0, 0)	0.11%	1 (0, 1, 0, 0)	0.00015%
BchL/ChlL	Cyanobacteria-like	111 (94, 88, 13, 30)	56.62%	91746 (72566, 18627, 68, 485)	76.57%
	Trebouxiophyceae-like	32 (2, 4, 23, 30)	15.46%	25715 (7, 22, 9867, 15819)	21.46%
	BchL UT 5	11 (0, 0, 11, 2)	5.31%	440 (0, 0, 412, 28)	0.37%
	BchL UT 4	6 (0, 1, 6, 1)	2.90%	1066 (0, 1, 1063, 2)	0.89%
	BchL UT 3	6 (0, 0, 6, 0)	2.90%	102 (0, 0, 102, 0)	0.085%
	*Phormidesmis priestleyi* Ana-like	5 (5, 2, 0, 0)	2.42%	270 (265, 5, 0, 0)	0.23%
	*Burkholderiales*-like	5 (2, 0, 4, 1)	2.42%	103 (5, 0, 97, 1)	0.086%
	*Bradyrhizobium* sp. S23321-like	4 (0, 4, 0, 0)	1.93%	146 (0, 146, 0, 0)	0.12%
	BchL UT 2	4 (4, 1, 3, 0)	1.93%	77 (6, 2, 69, 0)	0.064%
	*Sphingomonadales*-like	4 (2, 2, 2, 0)	1.93%	59 (40, 5, 14, 0)	0.049%
	*Sphingomonas*-like	3 (1, 3, 0, 0)	1.45%	10 (2, 8, 0, 0)	0.0084%
	*Kouleothrix aurantiaca*-like	3 (0, 3, 0, 0)	1.45%	8 (0, 8, 0, 0)	0.0067%
	*Methylobacterium*-like	2 (0, 1, 2, 1)	0.97%	12 (0, 2, 6, 4)	0.010%
	*Aquincola tertiaricarbonis*-like	2 (2, 0, 1, 0)	0.97%	12 (10, 0, 2, 0)	0.010%
	*Belnapia*-like	2 (2, 0, 0, 0)	0.97%	8 (8, 0, 0, 0)	0.0067%
	BchL UT 1	2 (0, 2, 0, 0)	0.97%	3 (0, 3, 0, 0)	0.0025%
	*Mesorhizobium*-like	1 (1, 0, 1, 1)	0.48%	27 (1, 0, 4, 22)	0.023%
	*Rhodovulum sulfidophilum*-like	1 (0, 1, 0, 0)	0.48%	1 (0, 1, 0, 0)	0.00083%
BchX	BchX UT 1	22 (0, 6, 22, 5)	47.83%	4453 (0, 34, 4125, 294)	89.96%
	*Methyloversatilis*-like	4 (0, 3, 1, 0)	8.70%	14 (0, 4, 10, 0)	0.28%
	*Bradyrhizobium* sp. S23321-like	3 (1, 3, 1, 1)	6.52%	300 (4, 293, 1, 2)	6.06%
	BchX UT 2	3 (0, 1, 3, 1)	6.52%	37 (0, 1, 26, 10)	0.75%
	*Novosphingobium acidiphilum*-like	3 (3, 1, 0, 0)	6.52%	30 (23, 7, 0, 0)	0.61%
	*Belnapia*-like	2 (0, 1, 2, 1)	4.35%	39 (0, 2, 32, 5)	0.79%
	*Methylobacterium*-like	1 (0, 0, 1, 0)	2.17%	42 (0, 0, 42, 0)	0.85%
	*Citromicrobium*-like	1 (0, 0, 1, 0)	2.17%	2 (0, 0, 2, 0)	0.040%
	*Bradyrhizobium*-like	1 (1, 1, 0, 0)	2.17%	2 (1, 1, 0, 0)	0.040%
	*Kouleothrix aurantiaca*-like	1 (0, 1, 0, 0)	2.17%	1 (0, 1, 0, 0)	0.020%
	*Methylocella silvestris*-like	1 (0, 1, 0, 0)	2.17%	1 (0, 1, 0, 0)	0.020%
	*Thiorhodospira sibirica*-like	1 (0, 1, 0, 0)	2.17%	1 (0, 1, 0, 0)	0.020%
	*Roseobacter denitrificans*-like	1 (1, 0, 0, 0)	2.17%	1 (1, 0, 0, 0)	0.020%

*Values in brackets refer to the OTUs and reads obtained from the individual samples KP2, KP15, KP43, and KP53, respectively. Separate OTUs without a close known cultured representative are not included. For the Cyanobacteria/Trebouxiophyceae-like ChlL cluster Cyanobacteria-like and Trebouxiophyceae-like sequences are listed separately*.

The PufM sequences appeared to be cosmopolitan: a broad diversity of cultured and uncultured sequences originating from habitats from all over the world, including polar regions, was found grouping with the new PufM sequences (Table S2). Some clusters, however, represented new PufM phylotypes, as no cultured or environmental PufM sequence was found grouping with them (e.g., cluster PufM UT 3).

The 119822 sequences that grouped with BchL/ChlL reference sequences constituted 207 OLUs, with the number of OLUs per sample varying from 66 to 114 (Table 3). A total of 48 OLUs contained only one sequence, whereas 96 OLUs were restricted to sequences from one sample (Figure S3, Table S1). Nine OLUs were common to all four samples (Figure S3). Of these, three OLUs (C0, C1, and C13) together represented 79.07% of all BchL/ChlL sequences (Table S1). OLU C13 (47.37% of sequences, mostly from KP2) and OLU C1 (13.38% of sequences, mostly from KP15) were ~97.7% similar to the ChlL sequences of *Synechococcus* sp. PCC 6312 (order *Chroococcales*, Cyanobacteria) and *Microcoleus vaginatus* (order *Oscillatoriales*, Cyanobacteria), respectively (Figure 1). Interestingly, OLU C0, containing 18.33% of the sequences and retrieved mostly from samples KP43 and KP53, displayed a very high similarity (~99.1%) to Trebouxiophyceae ChlL (Figure 1 and Figure S4, Table S1). All remaining OLUs contained less than 3.07% of the sequences.

Rarefaction analysis (Figure 2) showed saturation was not yet reached for the single, or the pooled samples, although the graphs started to flatten. Indeed, the estimated number of OLUs (Chao1 values) was only slightly higher than the number of observed OLUs, indicating most diversity was retrieved from the samples (Table 3). Furthermore, all samples showed a similar, very uneven distribution (Table 3).

**Figure 4:**
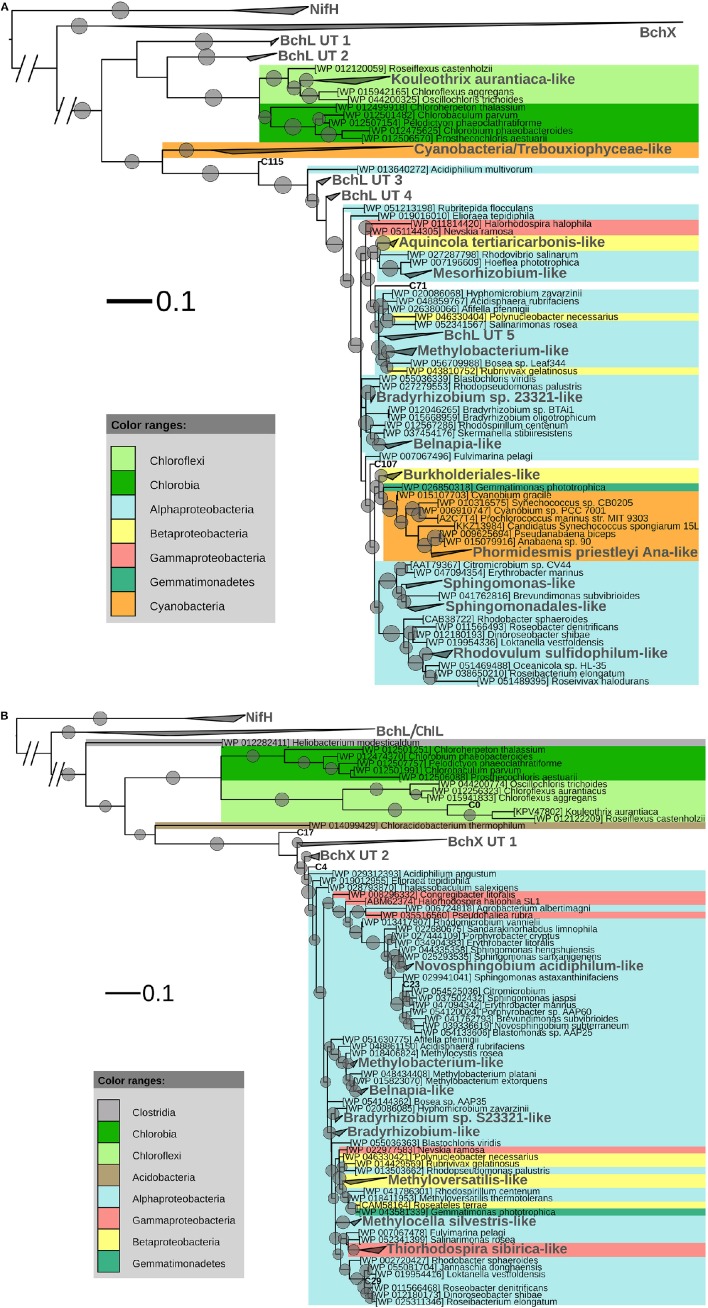
**(A)** ML phylogenetic tree (1000 bootstraps) of BchL/ChlL sequences. NifH sequences were used as an outgroup. Scale bar indicates 0.1 substitutions per amino acid position. OLU clusters (larger font size, labeled in gray) were named after cultivated bacteria that grouped in or close to the cluster. In the absence of cultivated members, clusters were given an Utsteinen (UT) cluster number designation. For clusters, the total branch lengths to the closest and farthest leaf of the cluster were used as sides of the triangle. OLUs not enclosed in clusters are labeled in bold. For reference data, taxon name and accession number is listed. Bootstrap values of at least 70% are displayed as circles with a diameter reflecting the height of the bootstrap value. BchX sequences are shown as a single cluster (details given in **B**). **(B)**. BchX ML phylogenetic tree (1000 bootstraps). NifH sequences were used as an outgroup. Scale bar indicates 0.1 substitutions per amino acid position. OXUs not enclosed in a cluster are labeled in bold. OXU clusters (larger font size, labeled in gray) were named after cultivated bacteria that grouped in or close to the cluster. In the absence of cultivated members, clusters were given an Utsteinen (UT) cluster number designation. For clusters, the total branch lengths to the closest and farthest leaf of the cluster were used as sides of the triangle. For reference data, taxon name, and accession number is listed. Bootstrap values of at least 70% are displayed as circles. Smallest circles represent the lower cut-off of 70% with a diameter reflecting the height of the bootstrap value. BchL/ChlL sequences are shown as a single cluster (details given in **A**).

After ML analysis, the 207 OLUs could be grouped into 17 clusters, 12 of which grouped with BchL/ChlL sequences from known microorganisms, and three separate OLUs (C71, C107, and C115) (Figures 1, 4A). The clusters were dispersed all over the BchL/ChlL phylogeny although the majority of OLUs and reads grouped with ChlL from oxygenic photosynthetic organisms (Cyanobacteria and Trebouxiophyceae green algae) (Table 4 and Table S1, Figures 1, 4A, and Figure S4). Notably, a small number of cyanobacterial reads from samples KP2 and KP15 (cluster *Phormidesmis priestleyi* Ana-like), together with sequences mainly obtained from marine unicellular Cyanobacteria (Figure 4A and Table 4), grouped among proteobacterial BchL. This aberrant grouping was previously ascribed to the occurrence of several shared conserved signature indels, absent from ChlL of other Cyanobacteria (Gupta, 2012). The other clusters grouped with BchL of Chloroflexi and Proteobacteria or belonged to four clusters (BchL UT 1–4) that could not be assigned to a named phylum (Figure 4A). OLUs grouping with Chloroflexi were only retrieved from sample KP15 (Figure 4A and Table 4). Of the clusters grouping among proteobacterial BchL, most grouped with Alphaproteobacteria, some with Betaproteobacteria, and none was found grouping with Gammaproteobacteria (Figure 4A).

No clear psychrophilic association could be deduced from the habitat metadata of the nearest neighbors. Most BchL/ChlL sequences grouped together with sequences retrieved from samples taken in a variety of ecosystems worldwide (Table S2).

A total of 4950 BchX sequences were obtained, many from sample KP43. They were binned at 95% protein similarity into 46 OXUs of which 31 were unique to one of the samples and 18 were singletons (Figure S3, Table 3, and Table S1). No OXU was found common between all four terrestrial samples (Figure S3). A single OXU, OXU C3, shared between samples KP15, KP43, and KP53, contained 82.10% of all BchX sequences and made up most of the reads retrieved from samples KP43 and KP53 (Figure 1, Table S1). However, phylogenetic analysis revealed that it did not group together with BchX sequences of known bacteria (Figure 4B). The less abundant OXUs C2 (3.68%) and C32 (2.61%) grouped in the same cluster as OXU C3 (BchX UT 1) and contained nearly exclusively sequences from sample KP43 (Table S1). OXU C1 (5.72%), containing sequences from samples KP2, KP15, and KP43, grouped with the BchX sequence originating from *Bradyrhizobium* sp. S23321 (Table S1). All other OXUs represented less than 1% of BchX sequences.

Rarefaction analysis (Figure 2) indicated that saturation was not reached. The graph of sample KP53, however, started to flatten. This was also confirmed by the number of estimated OXUs, which was nearly identical to the number of observed OXUs for sample KP53 (Table 3). Evenness analysis resulted in very low values, except for sample KP2, which showed a more even distribution, although this could be explained by the very low number of sequences retrieved from this sample, grouping in seven OXUs (Table 3).

After ML analysis, 27 OXUs, of which 22 grouped in cluster BchX UT 1, could not be associated with a named phylum. The remaining 19 OXUs grouped with BchX sequences of 11 known bacterial taxa (Figure 4B) of Chloroflexi and Alpha-, Beta-, and Gammaproteobacteria, although the latter was represented by only one sequence (OXU C30) (Figure 4B, Table S1). For samples KP43 and KP53, however, most of the sequences and OXUs grouped in two clusters (BchX UT 1 and BchX UT 2) or separate OXUs that could not be associated with a known BchX reference sequence (Figure 1, Table 4, and Table S1).

Similar to PufM and BchL/ChlL, no clear grouping of BchX sequences from cold habitats could be seen, as our sequences grouped with sequences retrieved from a broad diversity of terrestrial and aquatic ecosystems worldwide (Table S2).

## Discussion

Given the distance to the ocean (~200 km) and the extent of the surrounding ice cover, bacteria in exposed soils of the Sør Rondane Mountains, East Antarctica, are faced with very low availability of organic matter (Osanai et al., 2013; Zazovskaya et al., 2015; Tytgat et al., 2016) and might thus be expected to use alternative energy sources such as sunlight. During an initial survey using libraries of ~100 clones, phototrophy genes were investigated for the first time in this terrestrial Antarctic location (Tahon et al., 2016). Analysis of partial PufM sequences revealed diversity predominantly associated with phylotypes from aerobic anoxygenic photoheterotrophic Alphaproteobacteria. The most abundant ion pumping microbial rhodopsin gene family, proteorhodopsin, however, could not be amplified from the samples (Tahon et al., 2016). In the present study we used high-throughput Illumina MiSeq paired-end 300 bp sequencing to more comprehensively study the presence and diversity of genes involved in light-harvesting, in the same samples.

To target a wider diversity of anoxygenic phototrophs using the photosynthetic type 2 reaction center, the more universal pufM_uniF/pufM_WAW primer set (Yutin et al., 2005) was used. Using our *pufLM* database assembled from publicly available sequences, an *in silico* comparison of these primers with other less degenerate primers (i.e., pufM_557F, pufMR and pufM_750R) used in other studies (Nagashima et al., 1997; Achenbach et al., 2001) clearly showed that the Yutin et al. primer set (Yutin et al., 2005) targets a much wider *pufM* diversity (Figure S5). In addition, contrary to the clone library results (Tahon et al., 2016), the primer set used here gave successful amplification in all samples. As observed in the clone libraries, alphaproteobacterial-like PufM sequences were most frequently recovered (98.60%), followed distantly by gammaproteobacterial-like sequences (0.64%). Deep sequencing also revealed presence of some betaproteobacterial-like (0.012%) and even chloroflexi-like (0.38%) PufM (Figure 3). The dominance of alphaproteobacterial PufM sequences has been previously observed in the Arctic (Feng et al., 2014) and Antarctic (Karr et al., 2003; Koh et al., 2011), whereas chloroflexi-like PufM sequences have not previously been reported from polar or most other environments studied so far. Also in Arctic soils (Feng et al., 2014), gammaproteobacterial-like PufM sequences were found to contribute less to the general diversity.

The relative abundance of *Roseobacter*-like and *Loktanella*-like PufM in our samples is remarkably high (Figure 1). PufM from these two AAP-containing taxa have previously nearly exclusively been reported from marine and saline lake environments from polar and non-polar regions (Van Trappen et al., 2004; Oz et al., 2005; Du et al., 2006; Yutin et al., 2008; Cottrell and Kirchman, 2009; Jiang et al., 2010; Jeanthon et al., 2011; Koh et al., 2011; Ritchie and Johnson, 2012; Ferrera et al., 2014). The high relative abundance of *Roseobacter*-like PufM sequences (82.39%) in our terrestrial samples is therefore striking. *Roseobacter* has been found to be important in sulfur cycling in aquatic environments (González and Moran, 1997; Buchan et al., 2005; Wagner-Döbler and Biebl, 2006). However, the absence of *Roseobacter* 16S rRNA sequences in this study area (Tytgat et al., 2016) and in terrestrial Antarctic systems in general (based on metagenome data available in NCBI, MG-RAST, Wilke et al., 2016 and IMG/M Markowitz et al., 2014) suggests the presence of other microorganisms, containing PufM highly similar to that of *Roseobacter*, in our samples. The remainder of the PufM diversity in our samples, although recovered in small relative numbers, also mainly related to aquatic photoheterotrophic taxa. However, several of the PufM sequences recovered (e.g., *Methylobacterium*-like, *Rhodopseudomonas*-like) were highly similar to PufM reported from Arctic soils (Feng et al., 2014) or Chinese paddy soils (Feng et al., 2009, 2011a,b,c). Thus, although aerobic anoxygenic phototrophy is frequently studied in aquatic environments, our data strongly suggest that this lifestyle may potentially be important in terrestrial ecosystems.

The primers (Ando et al., 2005) previously used to amplify a broad diversity of *nifH* sequences also amplified structurally similar oxidoreductase subunits involved in (bacterio)chlorophyll synthesis (*bchL*/*chlL* encoding for the L subunit of DPOR in APB, Cyanobacteria, green algae, and lower land plants, and *bchX* encoding for a COR subunit of APB). The high relative abundance (98.26%) of cyanobacterial plus Trebouxiophyceae green algal ChlL suggested an important role for oxygenic photosynthetic organisms in our samples (Table 4, Figure 4A, and Figure S4). Notably, an inverse pattern was observed: nearly all of the ChlL reads recovered from samples KP2 (99.89%) and KP15 (98.92%) grouped with *Chroococcales* and *Oscillatoriales* Cyanobacteria, respectively (Table S1), with very few Trebouxiophyceae-like sequences (<0.12%). KP43 and KP53, on the other hand, contained far less cyanobacterial reads (0.58 and 2.96% respectively) and a very high relative abundance of Trebouxiophyceae-like ChlL (84.23 and 96.69% respectively) (Figure 1, Table S1). A similar pattern was observed previously (Tahon et al., 2016, under review): a high relative abundance of both cyanobacterial cbbL type IB (RuBisCO) and 16S rRNA, grouping with *Chroococcales* and *Oscillatoriales* Cyanobacteria, was recorded from the KP2 and KP15 samples, respectively, and much less in KP43 and KP53. A high relative number of trebouxiophyceael chloroplast 16S rRNA sequences was recorded from the latter samples (Tahon et al., under review).

However, as the primers were originally designed to amplify a broad diversity of *nifH* (Ando et al., 2005), and not *bchL*/*chlL*, it is conceivable that they might show a bias toward particular groups. Therefore, an *in silico* analysis, using a broad diversity of publicly available sequences, was performed to investigate possible primer bias. The IGK3 and DVV primers (Ando et al., 2005, Table 2) generally showed one or two mismatches, located at the 5′ primer end, with *bchL*/*chlL* reference data of different phyla (Figure S5). Chlorobi sequences, however, showed most mismatches (four and one in DVV and IGK3, respectively). The absence of clear differences in primer specificity for different groups suggests there is unlikely to be extensive bias in the primers. Thus, the high relative abundance of oxygenic photosynthetic microorganisms in ChlL suggests they indeed appear to be an important phototrophic group in the investigated samples.

In addition to the highly recovered cyanobacterial and green algal ChlL, a small number of non-cyanobacterial BchL sequences (1.74%–2091 sequences, including 25 singletons) grouped with a broad diversity of mainly aerobic anoxygenic phototrophic bacteria primarily belonging to alphaproteobacterial taxa and to lesser extent betaproteobacterial and chloroflexi taxa (Figure 4A).

The IGK3 and DVV primers also amplify *bchX*. Because this gene is not present in Cyanobacteria or in Trebouxiophyceae, the BchX dataset was relatively small (4950 sequences). The alternative explanation that primer mismatch might have reduced the number of sequences recovered, is less likely. Indeed, *in silico* analysis showed that the number of mismatches with a set of representative *bchX* sequences was limited (Figure S5). The low number of sequences precludes firm conclusions regarding BchX diversity. The BchX sequences mainly grouped with alphaproteobacterial taxa and to a lesser extent with Betaproteobacteria, Gammaproteobacteria, and Chloroflexi (Figure 4B), however, with greater sequencing depth, relative abundances may change. Most of the BchX reads grouped into two clusters (BchX UT 1 and 2) without any close known representative, indicating the existence of multiple not yet cultured or recognized APB. Indeed many anoxygenic phototrophs can grow heterotrophically and it is thus possible that some taxa, originally described as regular heterotrophs, may have phototrophic capacities that have not been noticed. Indeed, *Salinarimonas rosea* DSM 21201 originally tested negative for bacteriochlorophyll *a* synthesis (Liu et al., 2010) and the phototrophic capacities of *Skermanella stibiiresistens* SB22 were originally not reported (Luo et al., 2012), whereas more recent analysis of their genomic sequences (accession numbers AUBC01000000 and AVFL01000000) revealed their phototrophic potential.

Finally, since BchL/ChlL, BchX, and NifH exhibit a high degree of protein sequence similarity (Raymond et al., 2004), and their genes can be retrieved using the same primer set, it may be a challenge to correctly annotate these sequences. During our analyses, we noticed that public databases contain several BchL/ChlL and BchX sequences annotated as NifH, and *vice versa*. When studying these genes and including reference data, it is therefore important to take into account gene specific conserved amino acid positions to ensure correct interpretation of data.

Comparing the datasets for different genes is difficult as datasets obtained with different primers cannot be compared directly because of differences in primer specificity, PCR efficiency or bias. Nevertheless, because the large sampling depth of PufM complicates evaluation, we have normalized PufM and BchL/ChlL datasets (Table 3) to tentatively allow a rough comparison. This shows that pufM diversity is somewhat lower though of similar magnitude than that of BchL/ChlL (171 OPUs–192 OLUs). It should be noted that horizontal gene transfer may cause discrepancies between phylogenies of 16S rRNA or *cbbL*, and photosynthesis genes (Igarashi et al., 2000). However, tentatively, it can be noted that several of the APB taxa retrieved here were previously reported from 16S rRNA (e.g., *Bradyrhizobium, Sphingomonas, Afifella, Methylibium*) and *cbbL* (RuBisCO) (e.g., *Mesorhizobium, Bradyrhizobium, Methyloversatilis, Rhodospirillum centenum*) clone library and Illumina sequencing results from the same samples (Tahon et al., 2016, under review). Interestingly, the relative abundance of *Bradyrhizobium*-related sequences from sample KP15 was much higher than from the other samples in the three photosynthetic datasets (Table 4), as well as the 16S rRNA and cbbL datasets (Tahon et al., 2016) [Tahon et al., under review]. Furthermore, the genome of *Bradyrhizobium* sp. S23321—the closest neighbor to most of our *Bradyrhizobium*-related sequences—revealed a gene content adapted to survival in a broad range of environments (Okubo et al., 2006). The combination of these data thus suggests photoautotrophic bradyrhizobia may be present in sample KP15.

In the pilot study proteorhodopsin could not be detected (Tahon et al., 2016). Actinorhodopsin, a similar light-driven proton pump, was originally retrieved from aquatic Actinobacteria (Sharma et al., 2008) and little is known about its presence in terrestrial environments. Because Illumina data of partial 16S rRNA genes previously showed the samples investigated here to contain diverse Actinobacteria (6.26–23.48% of reads and 20.73–34.60% of OTUs, Tytgat et al., 2016) [Tahon et al., under review], we used several primer sets (Table 2) to amplify actinorhodopsin genes from our samples. While our attempts failed, this does not necessarily imply that these systems are absent in the terrestrial Antarctic bacterial communities. Currently available primers may be unsuitable to capture all actinorhodopsin diversity, as most reference data originates from aquatic systems (Sharma et al., 2008, 2009; Wurzbacher et al., 2012; Jezberová et al., 2013; Salka et al., 2014). Future metagenome datasets may resolve this question.

## Conclusions

We studied the presence of bacterial phototrophic pathways in a terrestrial Antarctic environment. While we could not detect actinorhodopsin genes, our analysis of other genes showed that a broad variety of oxygenic and anoxygenic phototrophs is present in soils of the Sør Rondane Mountains, East Antarctica. The high relative abundance of oxygenic photosynthetic microorganisms, however, suggests they are an important phototrophic group. Sequencing results of BchL, ChlL, and BchX, involved in (bacterio)chlorophyll synthesis, were dominated either by Cyanobacteria- or Trebouxiophyceae-related sequences. Moreover, the presence of currently unknown non-cyanobacterial phylotypes suggests the existence of multiple not yet cultured or recognized anoxygenic phototrophic bacteria. Illumina Miseq sequencing of PufM, typical for light-harvesting bacteria with a type 2 reaction center, revealed a very high relative abundance of two groups of sequences, i.e., *Roseobacter*-like and *Loktanella*-like, and a large diversity of other less abundant taxa from Alpha-, Beta-, and Gammaproteobacteria, Chloroflexi and several unassigned groups. Although photoheterotrophic bacterial light-harvesting is nearly exclusively studied in aquatic environments, our results suggest the potential relevance of this mechanism in terrestrial ecosystems.

## Author contributions

Conceived and designed the experiments: GT, AW. Performed the experiments: GT. Analyzed the data: GT, BT. Contributed analysis tools: BT. Wrote the paper: GT, AW. All authors approved the final manuscript.

### Conflict of interest statement

The authors declare that the research was conducted in the absence of any commercial or financial relationships that could be construed as a potential conflict of interest.

## Abbreviations

AAP: aerobic anoxygenic phototrophic bacteria
APB: anoxygenic phototrophic bacteria
COR: chlorin oxidoreductase
DPOR: dark-operative protochlorophyllide oxidoreductase
ML: maximum likelihood
OLU: operational ChlL/BchL unit
OPU: operational puf unit
OXU: operational BchX unit.
