# Supplementary material for: Diversity of Phototrophic Genes Suggests Multiple Bacteria May Be Able to Exploit Sunlight in Exposed Soils from the Sør Rondane Mountains, East Antarctica
Source: Front Microbiol. 2016 Dec 19;7:2026. doi: 10.3389/fmicb.2016.02026 (PMC5165242; doi:10.3389/fmicb.2016.02026)
Supplement: Supplementary file 1 [file DataSheet1.docx]

Supplementary Material

Diversity of Phototrophic Genes Suggests Multiple Bacteria May Be Able to Exploit Sunlight in Exposed Soils from the Sør Rondane Mountains, East Antarctica

Guillaume Tahon, Bjorn Tytgat, Anne Willems*

*** Correspondence:** Anne Willems: Anne.Willems@UGent.be

# Supplementary Figures and Tables

## Supplementary Figures


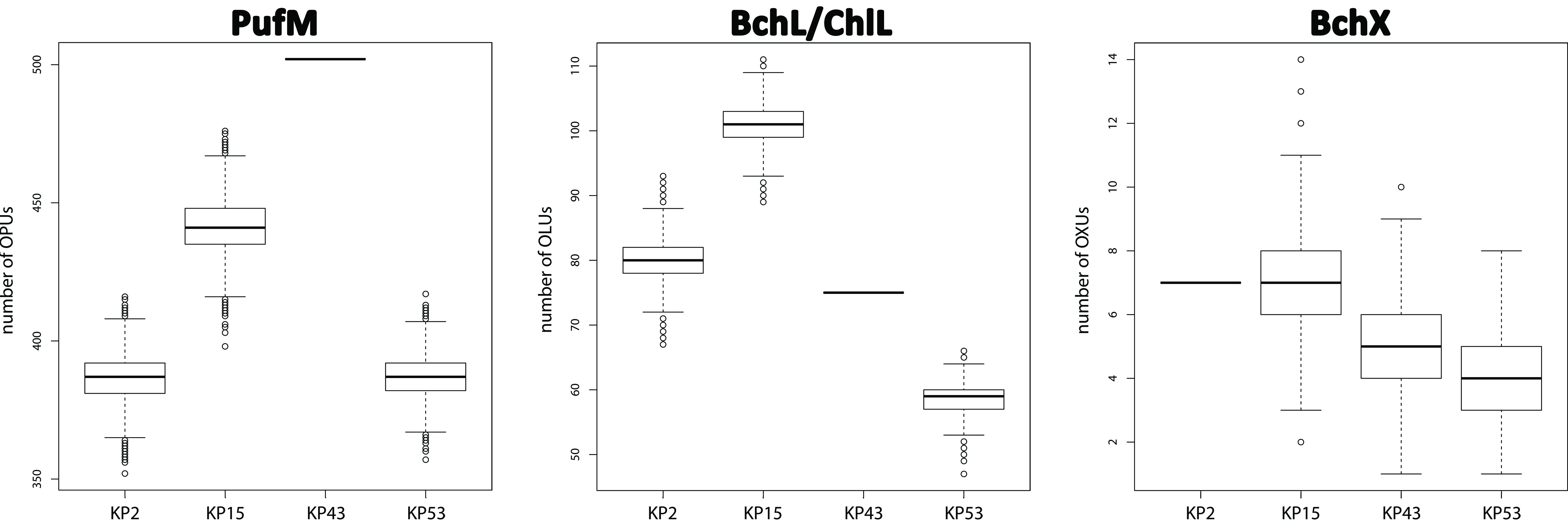


**Figure S1. Box plots showing variation in OPUs/OLUs/OXUs recovered after standardization.** For each gene, box plots were generated in R (<https://cran.r-project.org/>) using 10,000 iterations of the non-normalized OTU table.


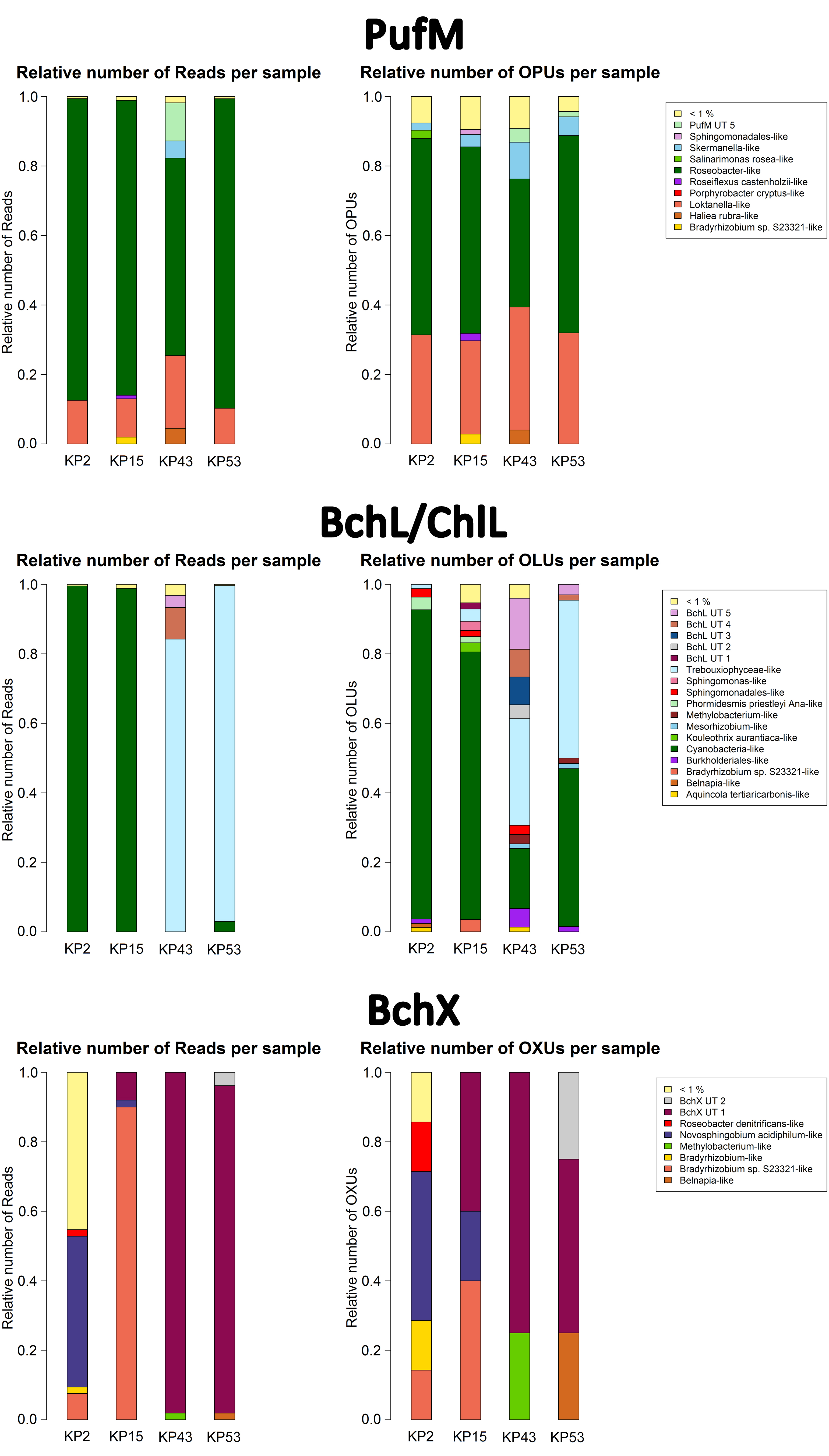


**Figure S2. Bar plots showing relative numbers of reads and OPUs (PufM), OLUs (BchL/ChlL) or OXUs (BchX) per cluster.** Data were normalized (non-normalized bar plots are shown in Figure 1). Clusters or separate OPUs/OLUs/OXUs containing less than 1% of the data were grouped together in the < 1% group.


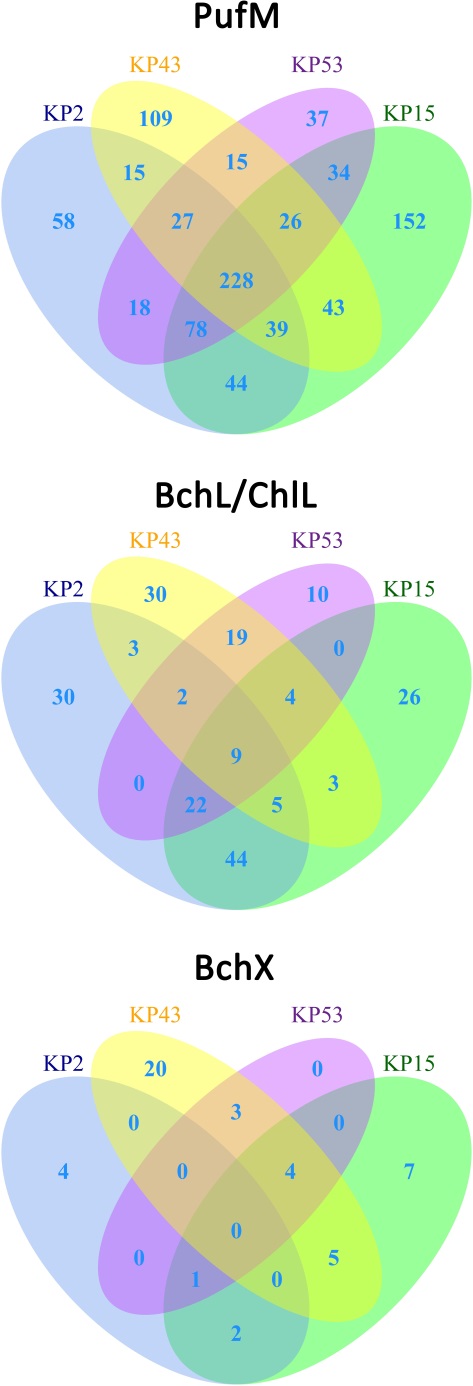


**Figure S3. Unweighted Venn diagrams of OPUs/OLUs/OXUs.** Venn diagrams were calculated using the VennDiagram package (<http://cran.r-project.org/web/packages/VennDiagram/index.html>) in R. Colored areas show the number of OPUs/OLUs/OXUs present in the terrestrial samples or shared between multiple samples.


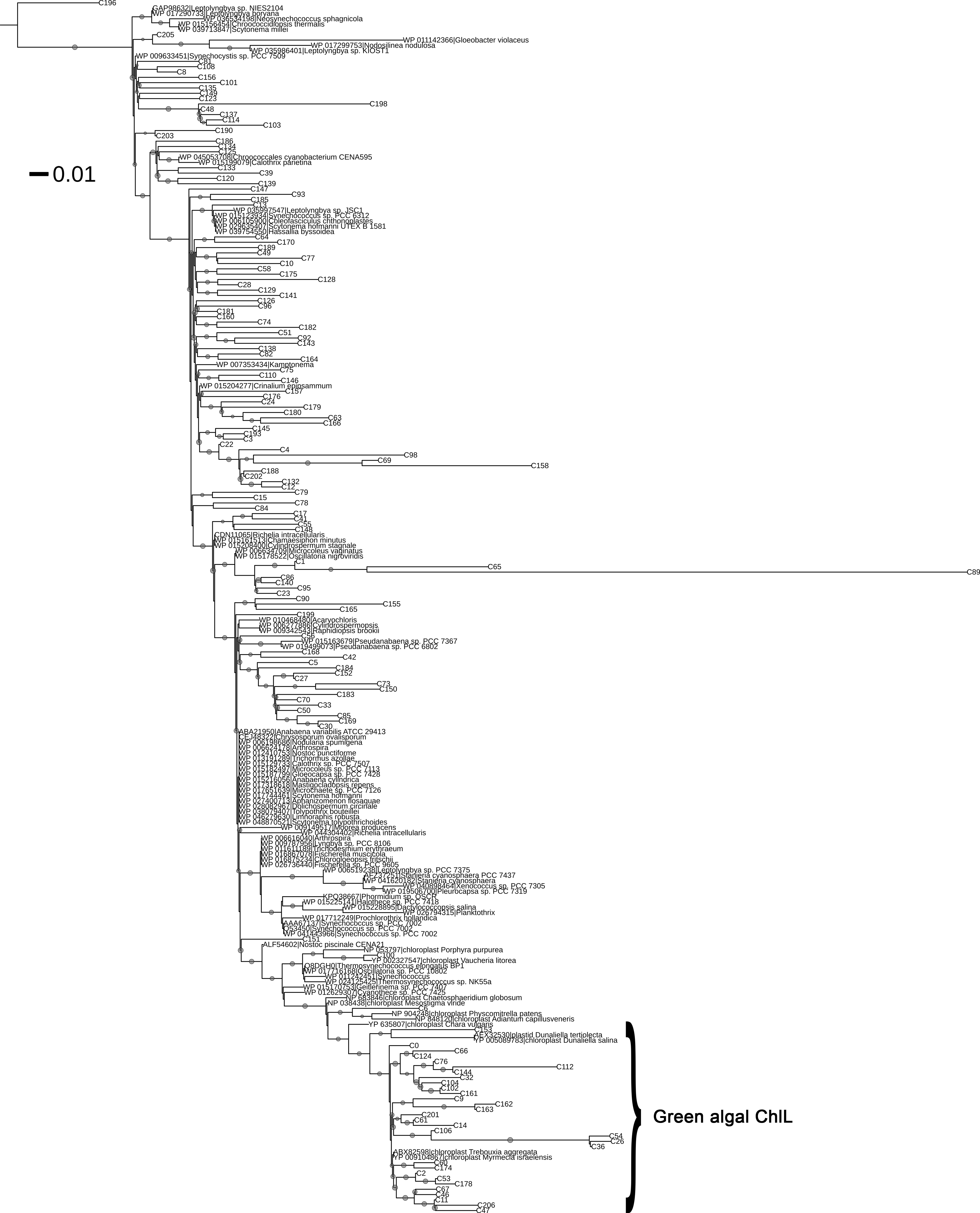


**Figure S4. Detailed view of the Cyanobacteria-like ChlL cluster from Figure 4A.** Scale bar indicates 0.01 substitutions per amino acid position. Bootstrap values of at least 70% are displayed as circles. Smallest circles represent the lower cut-off of 70% with a diameter reflecting the height of the bootstrap value.


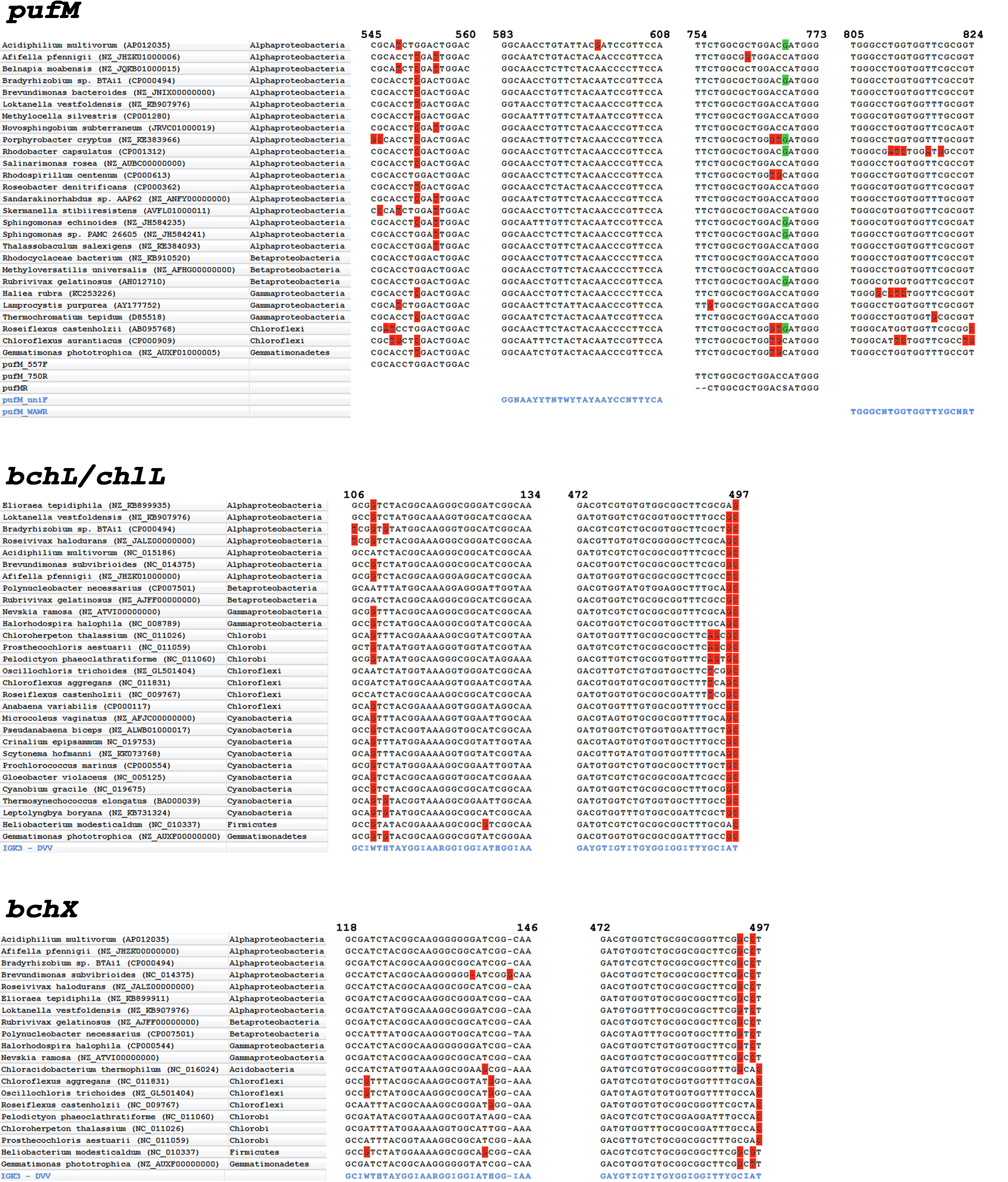


**Figure S5. Alignment of mismatches of *pufM*, *bchL*/*chlL* and *bchX* genes with primers used in this study.** Sequences of a selection of different phototrophic organisms are included. Accession numbers are given between brackets. Taxon (phylum or class) is given next to each organism name. Primers used are given in blue; for *pufM*, primers frequently used in literature are shown in black. Reverse primers are shown as reverse complementary sequences. Mismatches are labelled in red. For *pufM* primer pufM 750R, additional mismatches on position 768, compared to primer pufMR, are labelled in green. Nucleotide positions are relative to *Loktanella* *vestfoldensis* (NZ_KB907976).

## Supplementary Tables

File: Table S1.xlsx

**Table S1. Composition of OPUs, OLUs and OXUs.** Each worksheet is named after the gene analyzed and shows the number of sequences per sample for each OPU (PufM), OLU (ChlL/BchL) or OXU (BchX), as well as the cluster the OPU/OLU/OXU groups in. OPUs/OLUs/OXUs not enclosed in a cluster are labeled as Separate. For cyanobacterial OLUs, the order is given between brackets.

**Table S2. Habitat origin of nearest neighbors per named cluster, or separate OTU (95% amino acid similarity) with a close cultured representative.**

|  | **Cluster/Separate OTU** | **Closest neighbor(s)** | |
| --- | --- | --- | --- |
|  |  | **Accession no.** | **Origin** |
| PufM | *Acidisphaera* *rubrifaciens*-like | NZ_BANB01000656 | *Acidisphaera* *rubrifaciens*; Japan; Hot spring surface water and sediment |
|  | *Afifella*-like | NZ_JHZK01000006 | *Afifella* *pfennigii*; French Polynesia; Benthic microbial mat |
|  | *Belnapia*-like | NZ_KN676113 | *Belnapia* sp. F-4-1; China; Soil |
|  |  | NZ_JQKB01000015 | *Belnapia* *moabensis*; USA; Biological soil crust |
|  | *Bradyrhizobium* sp. S23321-like | AP012279 | *Bradyrhizobium* sp. S23321; Japan; Paddy field soil |
|  |  | JN248466 | ENV; Arctic ocean; Water (60 m deep) |
|  |  | KC768186 | ENV; Antarctica; Surface water |
|  |  | KF008147 | ENV; Antarctica; Surface water |
|  |  | AB510451 | ENV; China; Paddy soil |
|  |  | KM654597 | ENV; Norwegian and Barents sea; Water |
|  | *Brevundimonas*-like | NC_014375 | *Brevundimonas* *subvibrioides*; USA; Freshwater |
|  |  | NZ_JNIX01000000 | *Brevundimonas bacteroides*; USA; Freshwater |
|  | *Haliea rubra*-like | KC253226 | *Haliea* *rubra*; France; Surface water |
|  | *Hyphomicrobium*-like | NZ_KB911258 | *Hyphomicrobium* *zavarzinii*; Russia; Soil |
|  | *Ideonella*-like | NZ_BBYR01000007 | *Ideonella* sp. 201-F6; Japan; Sediment |
|  | *Loktanella*-like | NZ_KB907976 | *Loktanella* *vestfoldensis*; Antarctica; Lake microbial mat |
|  |  | JN378830 | *Loktanella* sp. RCC2403; Arctic ocean; Water (3 m deep) |
|  |  | JN248513 | ENV; Arctic ocean; Water (5 m deep) |
|  |  | KC900127 | ENV; Antarctica; Surface water |
|  |  | JF421745 | ENV; Mediterranean seawater |
|  |  | GU079560 | ENV; USA; Seawater |
|  |  | KF008169 | ENV; Antarctica; Surface water |
|  |  | GQ468949 | ENV; Mediterranean seawater |
|  | *Methylibium*-like | LGRD01000013 | *Methylibium* sp. NZG; China; *Bangia* *atropurpurea* |
|  |  | KC900142 | ENV; Antarctica; Surface water |
|  | *Methylobacterium*-like | NZ_KI912577 | *Methylobacterium* sp. 10; USA; Lake water |
|  |  | NZ_KB910516 | *Methylobacterium* sp. 77; USA; Lake water |
|  |  | NZ_AGJK00000000 | *Methylobacterium* *extorquens*; Finland; *Pinus* *sylvestris* |
|  |  | AP014705 | *Methylobacterium* *aquaticum*; Spain; Drinking water |
|  |  | NZ_JTHG01000063 | *Methylobacterium* *platani*; South Korea; *Platanus* *orientalis* leaf |
|  |  | JN248518 | ENV; Arctic ocean; Water (80 m deep) |
|  | *Methylocystis*-like | NZ_KB889963 | *Methylocystis* *rosea*; Norway; Wetland soil |
|  |  | NZ_AYNA01000017 | *Methylocystis* sp. SB2; USA; Spring bog |
|  |  | KF494876 | ENV; Norway; Seawater |
|  |  | AB510462 | ENV; China; Paddy soil |
|  | *Methyloversatilis*-like | NZ_AFHG00000000 | *Methyloversatilis* *universalis*; USA; Lake water |
|  |  | NZ_ARVV01000001 | *Methyloversatilis* *discipulorum*; USA; Lake water |
|  |  | NZ_KB889967 | *Methyloversatilis* *thermotolerans*; USA; Lake water |
|  | *Porphyrobacter cryptus*-like | NZ_KE383966 | *Porphyrobacter* *cryptus*; Portugal; Hot spring |
|  |  | AB510465 | ENV; China; Paddy soil |
|  | *Rhodopseudomonas*-like | NZ_LJIC01000210 | *Rhodopseudomonas* sp. AAP120; China; Freshwater lake |
|  | *Rhodospirillum* *centenum*-like | CP000613 | *Rhodospirillum* *centenum*; USA; Hot springs |
|  | *Rhodovulum* *sulfidophilum*-like | AB020784 | *Rhodovulum* *sulfidophilum*; Holland; Marine mud |
|  | *Roseicitreum* *antarcticum*-like | JQ995753 | *Roseicitreum* *antarcticum*; Antarctica; Sandy intertidal sediment |
|  |  | KF487001 | ENV; Norway; Water |
|  |  | KC900116 | ENV; Antarctica; Surface water |
|  | *Roseiflexus* *castenholzii*-like | AB095768 | *Roseiflexus* *castenholzii*; Japan; Microbial mat in hot spring |
|  | *Roseobacter*-like | CP000362 | *Roseobacter* *denitrificans*; Japan; *Enteromorpha* *linza* |
|  |  | CP002624 | *Roseobacter litoralis*; Seaweed |
|  |  | X57597 | *Erythrobacter* sp. OCH114 |
|  |  | NZ_JWLL01000010 | *Tateyamaria* sp. ANG-S1; USA; *Euprymna* *scolopes* |
|  |  | NZ_JYFE01000060 | *Jannaschia* *aquimarina*; South Korea; Seawater |
|  |  | JF421742 | ENV; Mediterranean seawater |
|  |  | JQ340526 | ENV; Pacific Ocean; Surface water |
|  |  | KM654595 | ENV; Norway; Seawater |
|  |  | AB510458 | ENV; China; Paddy soil |
|  | *Salinarimonas* *rosea*-like | NZ_AUBC00000000 | *Salinarimonas* *rosea*; China; Salt mine soil |
|  |  | KC900120 | ENV; Antarctica; Surface water |
|  | *Skermanella*-like | AVFL01000001 | *Skermanella* *stibiiresistens*; China; Iron mine soil |
|  |  | NZ_AVFK00000000 | *Skermanella aerolata*; South Korea; Air |
|  | *Sphingomonadales*-like | AYSC01000048 | *Blastomonas* sp. CACIA14H2; Brazil; Water |
|  |  | NZ_LJYW01000001 | *Prosthecomicrobium* *hirschii*; USA; Freshwater pond |
|  |  | NZ_ATVO01000008 | *Sandarakinorhabdus* *limnophila*; Germany; Freshwater lake |
|  |  | JRVC01000019 | *Novosphingobium* *subterraneum*; USA; Subsurface core (180 m deep) |
|  |  | NZ_KK073876 | *Sphingomonas* *jaspsi*; Japan; Freshwater |
|  |  | JQ340545 | ENV; Pacific Ocean; Surface water |
|  | *Sphingomonas*-like | NZ_JPJC00000000 | *Sphingomonas* sp. FUKUSWIS1; Germany; Lake water |
|  |  | NZ_JPDP00000000 | *Sphingomonas* sp. STIS6.2; Germany; Lake water |
|  | PufM UT 1 | JN248492 | ENV; Arctic ocean; Surface water |
|  | PufM UT 2 | JN248492 | ENV; Arctic ocean; Surface water |
|  | PufM UT 4 | JQ340684 | ENV; Pacific Ocean; Surface water |
|  |  | GQ468982 | ENV; Mediterranean seawater |
|  | PufM UT 5 | AB510461 | ENV; China; Paddy soil |
| BchL/ChlL | Cyanobacteria-like | ALF54602 | *Nostoc* *piscinale* CENA21; Brazil; Solimoes river |
|  |  | Q8DGH0 | *Thermosynechococcus* *elongatus* BP-1; Japan; Hot spring |
|  |  | WP_012410753 | *Nostoc* *punctiforme*; Australia; *Macrozamia* sp. root |
|  |  | WP_011142366 | *Gloeobacter* *violaceus*; Switzerland; Calcereous rock |
|  |  | WP_006634709 | *Microcoleus vaginatus*; USA ; Desert soil crust |
|  |  | WP_015204277 | *Crinalium epipsammum*; The Netherlands; Sandy crust |
|  |  | AIQ80516 | ENV; Glacier foreland soil |
|  |  | AIC84974 | ENV; USA; Strip mine lake |
|  |  | CEK40168 | ENV; Pakistan; Rhizosphere of *Pinus* *roxburghii* |
|  | Trebouxiophyceae-like | ABX82598 | chloroplast *Trebouxia* *aggregata* |
|  |  | YP_009104867 | chloroplast *Myrmecia* *israelensis* |
|  |  | AGZ19374 | chloroplast *Chlorella* sp. ArM0029B; Arctic |
|  |  | NP_045884 | chloroplast *Chlorella* *vulgaris*; water |
|  |  | YP_009104838 | chloroplast *Symbiochloris* *reticulata*; |
|  | BchL UT 7 | AIQ80532 | ENV; Glacier foreland soil |
|  | *Phormidesmis* *priestleyi* Ana-like | KPQ34041 | *Phormidesmis* *priestleyi* Ana; USA; Microbial mat |
|  | *Burkholderiales*-like | WP_056902525 | *Pseudorhodoferax* sp. Leaf274; Switzerland; *Arabidopsis* leaf |
|  |  | AGS08013 | *Aquincola tertiaricarbonis*; Germany; contaminated groundwater |
|  |  | AIC84916 | ENV; USA; Strip mine lake |
|  |  | AIQ80467 | ENV; Glacier foreland soil |
|  | *Bradyrhizobium* sp. S23321-like | WP_015684485 | *Bradyrhizobium* sp. S23321; Japan; Paddy field soil |
|  |  | ERF83826 | *Bradyrhizobium* sp. DFCI-1; USA; Human gut |
|  |  | AIQ80568 | ENV; Glacier foreland soil |
|  | *Sphingomonadales*-like | WP_022680698 | *Sandarakinorhabdus* *limnophila*; Germany, Lake water |
|  |  | WP_017667988 | *Sandarakinorhabdus* sp. AAP62; China; Lake water |
|  |  | WP_054135752 | *Blastomonas* sp. AAP25; Czech Republic; Lake water |
|  | *Sphingomonas*-like | WP_010405207 | *Sphingomonas* *echinoides*; Germany |
|  |  | WP_031439826 | *Sphingomonas* sp. FUKUSWIS1; Germany; Lake water |
|  |  | AIQ80498 | ENV; Glacier foreland soil |
|  | *Kouleothrix aurantiaca*-like | KPV49864 | *Kouleothrix* *aurantiaca*; Japan; Industrial waste water sludge |
|  | *Methylobacterium*-like | WP_003603754 | *Methylobacterium* *extorquens*; Finland; *Pinus* *sylvestris* |
|  |  | WP_048432787 | *Methylobacterium* *platani*; South Korea; *Platanus* *orientalis* leaf |
|  |  | WP_019903624 | *Methylobacterium* sp. 77; USA; Lake water |
|  |  | AIQ80515 | ENV; Glacier foreland soil |
|  | *Aquincola tertiaricarbonis*-like | WP_046114963 | *Aquincola tertiaricarbonis*; Germany; contaminated groundwater |
|  | *Belnapia*-like | WP_043338848 | *Belnapia* *moabensis*; USA; Biological soil crust |
|  |  | WP_043360866 | *Belnapia* sp. F-4-1; Tibet; Soil |
|  | *Mesorhizobium*-like | WP_027033752 | *Mesorhizobium* *loti*; Brazil; Zinc mine soil |
|  |  | AAY59338 | ENV; Canada; Soil sample near *Salix* *arctica* |
|  |  | AAY59364 | ENV; Canada; Rhizosphere of *Dryas* *integrifolia* |
|  | *Rhodovulum sulfidophilum*-like | WP_042458897 | *Rhodovulum* *sulfidophilum*; Holland; Marine mud |
|  |  | ALK25180 | ENV; Marine sample |
| BchX | *Methyloversatilis*-like | WP_054124830 | betaproteobacterium AAP99; China; Freshwater lake water |
|  |  | WP_020165691 | *Methyloversatilis* *discipulorum*; USA; Lake water |
|  |  | WP_008064756 | *Methyloversatilis* *universalis*; USA; Lake sediment |
|  | *Bradyrhizobium* sp. S23321-like | WP_015684462 | *Bradyrhizobium* sp. S23321; Japan; Paddy field soil |
|  |  | CEL12566 | ENV; Pakistan; Rhizosphere of wheat |
|  |  | AIQ80536 | ENV; Glacier foreland soil |
|  | *Novosphingobium acidiphilum*-like | WP_028640508 | *Novosphingobium* *acidiphilum*; Germany; Lake water |
|  | *Belnapia*-like | WP_052389091 | *Belnapia* *moabensis*; USA; Biological soil crust |
|  |  | AIC84959 | ENV; USA; Strip mine lake |
|  | *Methylobacterium*-like | WP_056467683 | *Methylobacterium* sp. Leaf104; Switzerland; *Arabidopsis* *thaliana* |
|  |  | WP_056479496 | *Methylobacterium* sp. Leaf117; Switzerland; *Arabidopsis* *thaliana* |
|  |  | WP_019903647 | *Methylobacterium* sp. 77; USA; Lake water |
|  | *Citromicrobium*-like | WP_054525036 | *Citromicrobium*; China; Seawater |
|  | *Bradyrhizobium*-like | WP_015668980 | *Bradyrhizobium* *oligotrophicum*; Japan; Rice field soil |
|  |  | WP_012046288 | *Bradyrhizobium* sp. BTAi1; USA; Stem nodules of *Aeschynomene* *indica* |
|  | *Kouleothrix aurantiaca­*-like | KPV47802 | *Kouleothrix* *aurantiaca*; Japan; Industrial waste water sludge |
|  | *Methylocella silvestris*-like | WP_012591062 | *Methylocella* *silvestris*; Germany; Acidic forest cambisol |
|  | *Thiorhodospira sibirica*-like | WP_006787504 | *Thiorhodospira* *sibirica*; Russia; Lake water |
|  |  | AIQ80570 | ENV; Glacier foreland soil |
|  | *Roseobacter denitrificans*-like | WP_011566468 | *Roseobacter* *denitrificans*; Japan; Fresh water |
|  |  | ALK25195 | ENV; Marine sample |
|  |  | AIC84928 | ENV; USA; Strip mine lake |
|  |  | AAT77530 | ENV; Mediterranean seawater |

For environmental studies, only one representative was used per cluster. ENV = environmental. For the Cyanobacteria/Trebouxiophyceae-like ChlL cluster Cyanobacteria-like and Trebouxiophyceae-like sequences are listed separately.
